# Inclusion of under-served groups in trials: an audit at a UK primary care clinical trials unit

**DOI:** 10.1186/s13063-025-08893-9

**Published:** 2025-06-21

**Authors:** Rebekah Burrow, Melanie Carr, Lucy Goddard, Lisa Hinton, Mike Clarke

**Affiliations:** 1 Nuffield Department of Primary Care Health Sciences, University of Oxford, Oxford, UK; 2 Queen’s University Belfast, Belfast, Northern Ireland UK

**Keywords:** Clinical trials, Inclusion, Under-served groups, Audit

## Abstract

**Background:**

Clinical trials need to include patients who are representative of the population who may receive the tested interventions in the future. The importance of inclusivity is recognised by ethical and funding bodies and has public support. Appropriate inclusion is required to provide equitable evidence-based healthcare and to comply with ethical principles for research. However, there is little information about the inclusivity of most under-served groups in UK clinical trials.

**Methods:**

This audit assesses the inclusion of under-served groups in trials run by the Oxford Primary Care Clinical Trials Unit (PC-CTU). We included trials with ethical approval between 2017 and 2023. We checked protocols, patient-facing information and selected data collection tools for information on the under-served groups in the INCLUDE guidance and protected characteristics in the UK Equality Act 2010, to identify explicit exclusions and data collection.

**Results:**

We included 19 trials. They were in a variety of clinical conditions, testing different types of interventions, both Clinical Trial of an Investigational Medicinal Product (CTIMP) and non-CTIMP. Most were non-commercially funded. We reviewed 21 protocols, 29 Patient Information Sheets/Leaflets and 40 data collection tools.

Common exclusions were based on age (19), sex or gender (11), language (8), capacity to consent (14), pregnancy (11), multiple health conditions (10) and severity of illness (17).

Trials most often collected data on age (19), sex or gender (15), ethnicity (16), education (11), address (13), mental health conditions (6), who gave consent (19), addiction (6), multiple health conditions (10), severity of illness (17), smoking status (12) and obesity (13).

**Conclusions:**

Often, exclusions were due to the focusing of the trial for a specific group, such as older people, women, or people being treated for a specific severity of condition. However, many explicit exclusions may not have been essential, may have reduced the inclusivity of the trials and might limit the applicability of the trial’s findings to people to whom the tested interventions might be relevant. These include the exclusion of people aged under 18, people without English language fluency and people without capacity to consent. All trials could have collected more informative data on under-served group status.

## Background

The findings of clinical trials can influence the care received by, and thereby the health of, people worldwide. To maximise the impact of clinical trials, trials need to recruit patients who are representative of the population who may receive the tested interventions in the future. This will support people making decisions about health care to feel confident about the applicability of the results. In the absence of clearly applicable evidence, interventions may be withheld or applied sub-optimally for people who are not represented in trials, contributing to health inequities for people in under-served groups [1–3]. 

Appropriate inclusion and active facilitation to participate are required to provide equitable evidence-based healthcare and to comply with ethical principles for research. Inclusivity is required to ensure that trials comply with Principle 13 of The Declaration of Helsinki: “Groups that are underrepresented in medical research should be provided appropriate access to participation in research” [4]. The impact of underrepresentation in research, on health outcomes, is clearly evidenced, for example, for people in minoritized ethnic groups and pregnant people during the COVID-19 pandemic [1, 5–9]. The importance of inclusivity is recognised by key UK ethical and funding bodies, including the NHS Health Research Authority (HRA), UK Research and Innovation (UKRI) and National Institute for Health and Care Research (NIHR) [10–12]. A recent survey by the NHS HRA found public support for diversity in health and social care research; although, of course, our individual and structural prejudices continue to have huge impacts on our society [13–15].

The NIHR is the largest UK funder of non-commercial research, and its strategy includes objectives to track and report diversity of research participants based on protected characteristics in the UK Equality Act 2010 [16]. The NIHR’s INCLUDE project described key characteristics of under-served groups and provided a non-exhaustive list of 34 under-served groups based on demographics, social and economic factors, health status and disease-specific factors [17]. Under-served groups are groups of people for whom the research community needs to provide a better service. These groups may have lower inclusion in research, a higher healthcare burden, and respond differently to healthcare interventions, with research neglecting to address these factors. A NIHR analysis of participation in a selection of their trials beginning between 2007 and 2017, by age, sex and ethnicity, found that representation was likely proportionate to the population of England and Wales, but that collection and reporting of these data requires improvement [18]. The NIHR is one of the key funders of trials carried out by clinical trials units (CTUs) in the UK. One of these CTUs is the Primary Care CTU (PC-CTU) at the University of Oxford, which provided the sample of trials for this audit.

There is little information about the current inclusivity of most under-served groups in UK clinical trials. A key aspect of inclusivity is explicit exclusion or inclusion for participants, as described in the trial protocol and patient-facing information. Implicit and subjective exclusions are also likely to be highly impactful but beyond the scope of this work [19–24]. We are aware of only one previous audit of the inclusivity of trials conducted by a UK-based CTU; at the Institute of Cancer Research Clinical Trials and Statistics Unit (ICR-CTSU) [25]. This audit included 30 trials approved between 2011 and 2021, examining first versions of the protocol, patient information sheet, questionnaire and first case report forms. The analysis focused on demographic factors, primarily sex and gender, and educational disadvantage, and compared statistically the results by date of approval and funding source, finding no significant impact of these.

The objective of our study was to assess inclusivity and the potential to assess participation of under-served groups in trials run by the PC-CTU in a potentially comparable and combinable way to Patel et al. [25].

We included trials led, managed and/or run by the PC-CTU, with first ethical approval between 2017 and 2023, to align with the second time window selected by Patel et al. [25], and to capture the most recent trials at PC-CTU. We reviewed protocols, patient-facing information, and selected data collection tools against all the under-served groups in the INCLUDE guidance and protected characteristics in the UK Equality Act 2010 [16]. We identified explicit exclusions of under-served groups in trial protocols and patient information and assessed whether data collection was sufficient to determine participation of under-served groups. The PC-CTU has used our findings to establish an inclusivity working group to prioritise and coordinate efforts to improve inclusivity of, and data collection on, under-served groups, and to support other CTUs to undertake similar audits.

## Methods

The PC-CTU, established in 2001, is a world-leading centre for primary care clinical trials with clinical priorities of infectious diseases, cardiovascular and metabolic disease and behavioural medicine [26]. It is part of the UK Clinical Research Collaboration (UKCRC) fully registered Oxford Primary Care and Vaccine Collaborative Clinical Trials Unit [27].

### Included trials

All trials led, managed and run by the PC-CTU and sponsored by the University of Oxford, with first ethical approval of a study protocol between 1 January 2017 and 31 December 2023, were included in the audit.

### Included documentation

For these trials, documentation requested from trialists were: first version of the protocol with ethical approval (and last version, including appendices, for platform trials), participant information sheets (PIS), all templates for data-collection questionnaires and case report forms (CRFs), and all other forms that collected data (which might indicate whether a participant was in an under-served group and had protected characteristics), and templates for consent forms where assent or consent by a legal guardian or representative could be given. Only the first version of the protocol with ethical approval, for trials that were not platform trials, was included, in common with the methods used by Patel et al. [25], after an exploration of multiple versions for two trials yielded no additional eligibility criteria, and based on advice from CTU colleagues that the eligibility criteria for the 16 trials were very unlikely to have changed in this respect.

### Documentation review

We reviewed documents for items which could be mapped onto the INCLUDE under-served groups and/or protected characteristics in the UK Equality Act 2010. Protocols and PIS were searched for explicit exclusions of under-served groups and/or protected characteristics. Only explicit exclusions were included. For example, if a trial carried out recruitment only through GP practices, we did not infer that this was an exclusion of people who do not attend regular medical appointments. Questionnaires, CRFs, administrative forms and consent forms were searched for explicit collection of data identifying participants as members of an under-served group or as having a protected characteristic. Questionnaires, CRFs, administrative forms that did not include explicit exclusions or data collection were not included in the audit. Only explicit data were included. For example, if a trial included an EQ-5D instrument we did not assume that data collected in the anxiety/depression dimension could be used to assess whether participants had these mental health conditions. One researcher (RB) searched all documents, extracted information and coded it. Another researcher (LG) checked and verified the search, extraction and coding. Discrepancies were resolved through re-examination and discussion. Inter-rater reliability metrics were not calculated.

### Counting documents and exclusions

Where trials had more than one protocol, PIS, or data collection tool, these were all assessed. An overall interpretation was made for each trial for each category (protocol, patient-facing information and data collection) only counting exclusions if they applied throughout the trial. When an exclusion could be counted in more than one under-served group (for example, a visual or hearing impairment is a physical disability and both people with these impairments and those with a physical disability are under-served groups listed in the INCLUDE guidance), it was counted once in the most detailed under-served group (in this case, visually or hearing impaired). When more than one clinical condition was relevant to a trial, the single condition most relevant to the condition for which participants were included in the trial was selected (for example, a trial of sleep support for people with insomnia and depression was classed as “Sleep” rather than “Psychiatric”). This ensured the most conservative method of assessing exclusion was used to generate a “best-case” scenario of inclusivity. We did not assess whether exclusions might be appropriate, necessary, or ethical.

Summary statistics were used to describe the characteristics of the trials, inclusivity of protocols and PIS and completeness of data collected. No statistical correction methods were used. No sensitivity analysis was conducted. Excel was used for data analysis.

## Results

We included 19 trials in the audit; their characteristics are described in Table 1. We reviewed 21 protocols, 29 PIS/PIL and 40 data collection tools.

**Table 1:** Trial characteristics

Clinical condition	
Infection	8
Endocrinology	2
Obstetric	2
Sleep	2
Cardiovascular	1
Nephrology	1
Neurology	1
Psychiatric	1
Respiratory	1
CTIMP	
non-CTIMP	12
CTIMP	7
Intervention	
Drug	6
Diagnostic	3
Monitoring or prognostic	5
Technology	3
Support	2
Phase	
I	1
II	0
III	2
IV	4
Funding	
Commercial	2
Non-commercial	17

Support—these were trials of additional support and guidance for participants by clinicians

*CTIMP* Clinical Trial of an Investigational Medicinal Product

Three infections trials were COVID-19 platform trials. One endocrinology trial was a feasibility trial for another trial that was also included in the audit. One infections trial was stopped before recruitment began, and a subsequent trial that was based on this was also included in the audit.

### Demographic factors

#### Age

Protocols for all trials specified a lower age limit, 1 year (1), 12 years (1), 18 years (11), 35 years (2), 50 years (1), 60 years (2), or 75 years (1). One protocol specified an upper age limit of 74 years. Fourteen trials had PIS that specified a lower age limit; one specified an upper age limit. Some trials communicated slightly different limits in the PIS than in the protocol (for example, “aged over 18” compared to “aged 18 years or above”). All trials collected data on age, 12 collected date of birth, 6 collected age in years and one collected whether the participant’s age was within the eligible range.

**Fig. 1:**
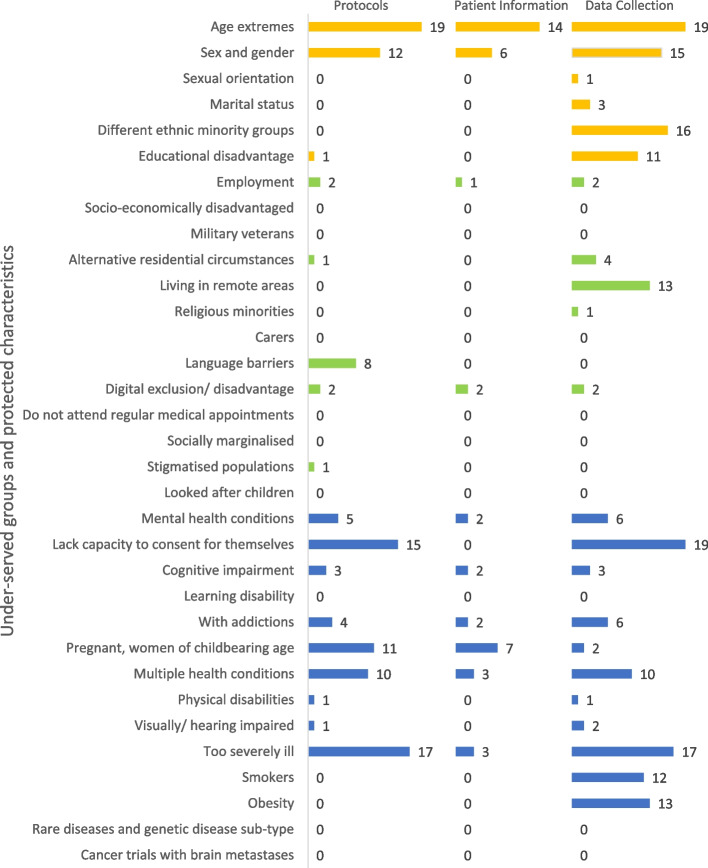
Trials with explicit exclusions of, and collecting data on, under-served groups and protected characteristics

Figure 1 key: Demographic factors are shown in orange, social and economic factors in green, health status in blue; there were no visible data points for “disease-specific factors”.

#### Sex or gender

Protocols for 12 trials specified inclusion criteria based on sex or gender using terms such as “women”, “female”, or “male”. None specified whether they meant sex/gender assigned at birth or gender identity, although this could be presumed to be sex assigned at birth for six trials during pregnancy or of urinary tract infections (UTIs) including only “female” or “women”. These six trials also specified in the PIS that participants were women. One trial used “any gender” as an inclusion criterion in the protocol. There were no explicit exclusions of people with a gender identity different from the sex assigned to them at birth, or who had or were undergoing gender reassignment. Five trials collected data on gender, seven collected data on sex; one of these trials also collected gender assigned at birth and gender identified, three of these trials collected sex at, or assigned at, birth. Three other trials collected data on sex/gender but did not specify whether data was sex or gender.

#### Sexual orientation, marital/civil partnership status

No trials had explicit restrictions on participants’ sexual orientation. One trial collected data on sexual orientation. There were no trials with restrictions on the marital status or civil partnership of participants. Three trials collected data on marital status, but none of these sought data relating to civil partnership.

#### Ethnicity or race

There were no trials with restrictions on ethnicity. Sixteen trials collected data on ethnicity. These 16 trials used nine different sets of potential answers to collect information about ethnicity; one trial had a set of four potential closed answers, while the others had as many as 18 options with multiple opportunities for free text description.

#### Education

One protocol had an exclusion criterion that required participants to have a minimum of seven years of formal education. Eleven trials collected information about the level of education participants had achieved.

### Social and economic factors

#### Employment

Participants were excluded from two trials testing interventions for insomnia because of their working hours; exclusions were documented in protocols (2 trials) and PIS (1 trial). One of these trials and one other trial collected data about employment status.

#### Socio-economic status

There were no trials with eligibility criteria based on socioeconomic status (SES), and no trials collected data that could be used to assess this with certainty. However, most trials collected data that might be used to estimate SES, such as postcode.

#### Military veterans

No trials had restrictions relating to military service based on their protocols or PIS, and none collected data on this.

#### Alternative residential circumstances

One trial explicitly excluded nursing home residents in its protocol, and four trials collected data on some residential circumstances.

#### Remote areas

No trials provided information on whether people in remote areas were eligible or ineligible. One trial communicated different (partially overlapping) eligible geographical areas—“Oxfordshire”, compared to “Thames Valley”. Thirteen trials explicitly collected participant address and/or a partial or full postcode. The other six trials may have collected this information in an administrative database or held the data only at trial sites; if so, these data were not accessible for this audit.

#### Religion and belief

There were no trials with restrictions on religion or belief. One trial collected data on religion.

#### Carers

There were no trials with restrictions on whether participants could be a carer, and none collected data on whether participants were carers.

#### Language barriers

Seven trials required a level of fluency in English in the eligibility criteria in their protocols. An additional multi-country study required participants to have an understanding of “the local language”.

#### Digital disadvantage

Two trials, a feasibility trial and its subsequent full trial, included a digital intervention. These trials required participants to have access to a mobile phone (according to the PIS) and the ability to send, understand and retrieve text-messages (according to the protocol). These trials collected data on mobile phone and other digital device use.

#### Do not attend regular medical appointments

There were no trials with explicit restrictions on people who do not attend regular medical appointments, and none collected data that would reveal participation.

#### Socially marginalised people

There were no trials with restrictions on socially marginalised people, and none collected data on whether participants were socially marginalised.

#### Stigmatised populations

The protocol for one trial, in a condition unrelated to tuberculosis, excluded the majority of patients with tuberculosis. No data was collected on this or other stigmatised populations.

#### Children in care

There were no trials with restrictions on looked-after children (children in care). In the two trials that included participants under 18 years of age, no data was collected on whether participants were children in care.

### Health status

#### Mental health conditions

The protocols for five trials explicitly excluded people with selected mental health conditions; two of these trials were for people with other specific mental health conditions, and they also included some of this information in their PIS. Six trials collected data on mental health conditions.

#### Capacity to consent

Through our review of protocols, 14 trials excluded all people without capacity to consent. The two trials including people aged under 18 years enabled the participation of children through assent and consent by a legal guardian. One of these also made provision for a legal representative to provide consent for at least some adults without capacity to consent for themselves. Three other trials made provision for a legal representative to provide consent for at least some adults without capacity to consent for themselves. All 19 trials collected data on who had provided consent.

#### Cognitive impairment

One trial for participants with mild cognitive impairment, one trial in mental health, and one trial in sleep had exclusions of people with severe (one) or any (two) cognitive impairment in their protocols. Two of these included this information in their PIS. Two of these trials and one other trial collected data on cognitive impairment.

#### Learning disabilities

There were no trials with explicit restrictions on learning disabilities, and none collected data on whether participants had learning disabilities.

#### Addictions

Four trials excluded people with addictions to alcohol or other drugs or substances. Two included this information in their PIS. Three of these trials, and another three trials, collected information about alcohol or other drugs or substances use.

#### Pregnancy

One trial excluded premenopausal women in the protocol. Eight other trials excluded people during pregnancy, of which six also excluded people who were planning a pregnancy. Four of these eight trials excluded people who were breastfeeding or lactating, and two other trials excluded people who had been pregnant in the last three months. Seven of these 10 trials included some of this information in the PIS. Two trials were obstetric trials including people at a specific stage of pregnancy; this information was in both the protocol and the PIS. These two trials and one other collected data on pregnancy.

#### Multiple health conditions

Ten trials excluded people with specific multiple health conditions, three included some of this information in the PIS. Three other trials were designed to focus on participants with multiple health conditions and also included this information in the PIS. Ten trials collected data on selected health conditions, ranging from three specified conditions to the use of the Cambridge Multimorbidity Score.

#### Physical disabilities, visual or hearing impairment

One trial excluded people with any of a wide range of disabilities; this information was only in the protocol. This trial also specifically excluded people with visual or hearing impairments. One trial collected data on whether participants were registered as disabled and used the Cambridge Multimorbidity Score to collect information about visual and hearing impairments. One trial collected data about problems experienced because of visual or hearing impairments.

#### Too severely ill

Seventeen trials excluded patients who were too severely ill with the condition under study. This was due to the community or primary care setting of the trial (seven), the medications the patient would require (nine) or their life expectancy (five), amongst other reasons. This information was included in three PIS. All 17 of these trials collected data on severity of the condition under study, through a variety of frequency, severity and duration of episodes, signs, and symptoms, and medication use. Two trials were designed to assess the new onset of a condition.

#### Smoking and obesity

There were no trials with explicit restrictions on participants who smoke or with obesity. Twelve trials collected data on smoking status. Eleven of these trials and two others collected data on obesity.

### Disease-specific factors

No trials in our audit were of participants with rare diseases or genetic disease sub-types, and none were cancer trials.

### Inclusion of under-served groups

Eleven trials were designed to focus on specific groups who are under-served by trials in general, including women, people during pregnancy, people with mental health conditions, older people and people with multiple health conditions. For these, some eligibility criteria relate to selecting the appropriate population within the under-served group. For example, a trial during a specific stage of pregnancy must exclude people at other stages of pregnancy. Generating evidence for one under-served group can mean excluding another under-served group; for example, a trial in people over 75 necessarily excludes people in the under-served age group (< 18 years).

At least seven trials had made deliberate attempts to include some under-served groups even when they were not focusing on these groups. Examples are the inclusion of children, allowing for consent by a legal representative, the use of gender-inclusive language, provision of patient information in different languages, and platform trials with additional arm-specific exclusions to enable participation in at least some of the trial’s treatment comparisons.

## Discussion

This is only the second audit of which we are aware, that has examined the inclusivity of trials being run by a UK CTU. Explicit exclusions of people from under-served groups or those with protected characteristics differ between many of the 19 trials in this sample. Trial protocols and PIS may need to become more explicit about the potential to include these people in the trial and to collect data on their participation to show the inclusivity of clinical trials in the UK. Greater detail in the communication of eligibility criteria (for example, age, sex and gender, geographic location) in patient-facing information could help improve recruitment and efficiency by indicating which often excluded groups are included, and informing other potential participants at an earlier stage that they are not eligible. Greater consistency in data collection would help in describing the people who take part in these trials. For instance, there was a lack of clarity about what was being collected (for example, sex or gender), widely varying categories were used (for example, ethnicity) and some common options were missing from set answers (for example, marital status). Consistency in data collection would also support meta-analysis considering under-served groups. We found many examples of good practice by the PC-CTU in the focus on, and inclusion of, specific under-served groups and collection of data on participation of under-served groups. Wider adoption of these exemplars and the following of existing external guidance for inclusive research might increase inclusivity of, and assessment of participation in, trials.

Although many explicit exclusions were due to focusing a trial on a specific population, some explicit exclusions may have been directed by resource constraints, such as exclusion of under 18-year-olds, English language fluency, and capacity to consent. Furthermore, some exclusions extended beyond the focused group. For example, criteria designed to exclude people during pregnancy or breastfeeding sometimes also excluded people not willing to use specific forms of contraception.

Based on these findings, an inclusivity working group has been established within the PC-CTU to prioritise and coordinate efforts to improve inclusivity of, and data collection on, under-served groups. At the time of writing, seven more registered CTUs in the UK have initiated similar coordinated audits. There are many available frameworks and tools to support researchers in trying to make trials more inclusive [19, 28–33].

### Limitations

We included only trials led, managed and run by the PC-CTU, based in the University of Oxford in England. This may limit the representativeness of our sample of trials, and it would be interesting to examine a larger, more diverse sample of trials. It would also be interesting to investigate the actual recruitment of people from under-served groups and those with protected characteristics where these are not explicitly excluded from the trials.

The audit date range includes the beginning of the COVID-19 pandemic, which had a huge impact on the type, size and number of trials conducted by the PC-CTU, and the resources available for non-COVID trials. An updated audit, including trials from after the pandemic, might provide different results.

We did not assess exclusions for appropriateness. For example, we did not assess whether trials excluding people under 18 were of conditions that occur in people under 18. Exclusions may be necessary and ethical; for example, exclusion of people during pregnancy from a trial of a known teratogen.

Our audit considered only explicit exclusions, and conservatively counted exclusions per under-served group or protected characteristic. Inclusivity of clinical trials is determined by many factors; for example, trial recruitment strategies, lack of trust in research, inequalities within service access, all of which, and many more, would all have a large influence on inclusivity of trials [34–36]. Implicit and subjective exclusions not considered in this audit are likely to play a significant role in the underrepresentation of under-served groups in research. Many of the trials in the PC-CTU are funded by the NIHR, which has a clear strategy for, and is leading a drive for, inclusivity in trials. This means that inclusivity of people in under-served groups in UK clinical trials more generally might be lower than in the PC-CTU, although this will need further study.

The examples of under-served groups listed in the NIHR INCLUDE guidance are just that, examples. The guidance is clear that under-served groups are context and study specific, and the list here is not exhaustive. We may have considered under-served groups that are not under-served in these trials or overlooked under-served groups that are. In addition, retrospectively applying these groups to criteria and data collection was often a subjective process. The prospective consideration of under-served groups, inclusivity, and data collection to assess participation when designing trials is preferable.

## Conclusions

Appropriate inclusion and active facilitation to participate in clinical trials are required to comply with ethical principles for research and to provide knowledge that will support equitable evidence-based healthcare after the trials. This is the second audit of inclusivity of trials of a UK academic trials unit, and it shows that some trials recently conducted by the PC-CTU made specific efforts to include some under-served groups and enable assessment of their participation. However, more attention needs to be given to include more under-served groups, make inclusions explicit, and to collect better data to assess participation.

## Data Availability

The audit data that support the findings of this study will be made available for research that sets out to achieve aims specified in a methodologically and scientifically sound protocol approved by the University of Oxford Nuffield Department of Primary Care Hosted Research Datasets Committee (PrimDISC). Information regarding how to apply for access to these data should be directed to: primdisc@phc.ox.ac.uk.

## Abbreviations

CRF: Case report form
CTIMP: Clinical Trial of an Investigational Medicinal Product
CTU: Clinical Trials Unit
HRA: Health Research Authority
NIHR: National Institute for Health and Care Research
ICR-CTSU: Institute of Cancer Research Clinical Trials and Statistics Unit
PC-CTU: Primary Care Clinical Trials Unit
PIS: Patient Information Sheet
SES: Socioeconomic status
UKCRC: UK Clinical Research Collaboration
UKRI: UK Research and Innovation
UTI: Urinary tract infection
